# Numerical study on the biomechanics mechanism of Type II endoleak after EVAR for Abdominal Aortic Aneurysm

**DOI:** 10.1371/journal.pone.0323358

**Published:** 2025-05-28

**Authors:** Xiao Mo, Feng Zhang, Kai-Xiong Qing, Yang Xiao

**Affiliations:** 1 Faculty of Information Engineering and Automation, Kunming University of Science and Technology, Kunming, Yunnan, China; 2 Department of Cardiovascular Surgery, The First Affiliated Hospital of Kunming Medical University, Kunming, Yunnan, China; Coventry University, UNITED KINGDOM OF GREAT BRITAIN AND NORTHERN IRELAND

## Abstract

Type II endoleak, a common complication following Endovascular Aneurysm Repair (EVAR), remains a leading cause of re-intervention, yet its biomechanical mechanisms are not fully understood. This study employed an idealized model of the aneurysmal sac, Inferior Mesenteric Artery (IMA), and Lumbar Arteries (LAs) to explore endoleak mechanisms using Computational Fluid Dynamics (CFD) and Fluid-structure Interaction (FSI) simulations. The results demonstrated that pressure differences between the sac and branch arteries drive Type II endoleak, with the IMA often serving as the primary inlet. Under the influence of Type II endoleak, flow disturbances near the IMA and LAs suggest potential challenges in thrombus formation. Comparative analysis between CFD and FSI showed that CFD overestimated peak flow velocity in the IMA and underestimated sac pressure, while also displaying a temporal lag in velocity and pressure curve throughout the cardiac cycle. These findings indicate that CFD is suitable for rapid assessments where precision is less critical, whereas FSI is more appropriate for detailed mechanistic studies. This work clarifies the biomechanics of Type II endoleak and underscores the importance of selecting appropriate numerical methods in clinical and research contexts.

## 1 Introduction

Abdominal Aortic Aneurysm (AAA), characterized by localized arterial wall dilation, is the most common type of aneurysm. Despite emergency surgery, ruptured AAA cases exhibit high mortality rates [1–3]. Endovascular Aneurysm Repair (EVAR) has emerged as a preferred treatment due to its minimally invasive approach and faster recovery [4–7]. However, complications such as Type II endoleak, caused by retrograde blood flow into the aneurysmal sac through branches like the Lumbar Arteries (LAs) or Inferior Mesenteric Artery (IMA), remain a leading cause of re-intervention [8–10].

Clinical studies have primarily relied on statistical analyses of endoleak incidence and intervention outcomes, yet they fail to elucidate the biomechanical mechanisms underlying Type II endoleaks [11–14]. These mechanisms are closely tied to factors such as branch vessel pressure, vessel diameter, blood viscosity, vascular wall properties, and shear stress. However, obtaining these parameters clinically is highly challenging and often impractical.

Recent advancements in computational methods have made numerical simulation a key tool in AAA biomechanical research. While Computational Fluid Dynamics (CFD) is widely used for its efficiency, Fluid-Structure Interaction (FSI) offers greater accuracy by accounting for wall deformation, albeit at higher computational costs [15–20]. Comparative studies have shown that incorporating wall elasticity in FSI improves simulation precision under similar conditions [21,22]. However, the differences between CFD and FSI in studying Type II endoleak mechanisms remain unexplored.

To address this gap, this paper develops an idealized AAA model using both CFD and FSI to investigate blood flow patterns within the aneurysmal sac under transient branch vessel pressures, aiming to uncover the mechanisms behind Type II endoleak. Additionally, through a comparative analysis of CFD and FSI, we quantitatively highlight their differences, clarifying potential biases introduced by CFD-only approaches.

## 2 Materials and methods

### 2.1 Model and mesh

**Fig 1:**
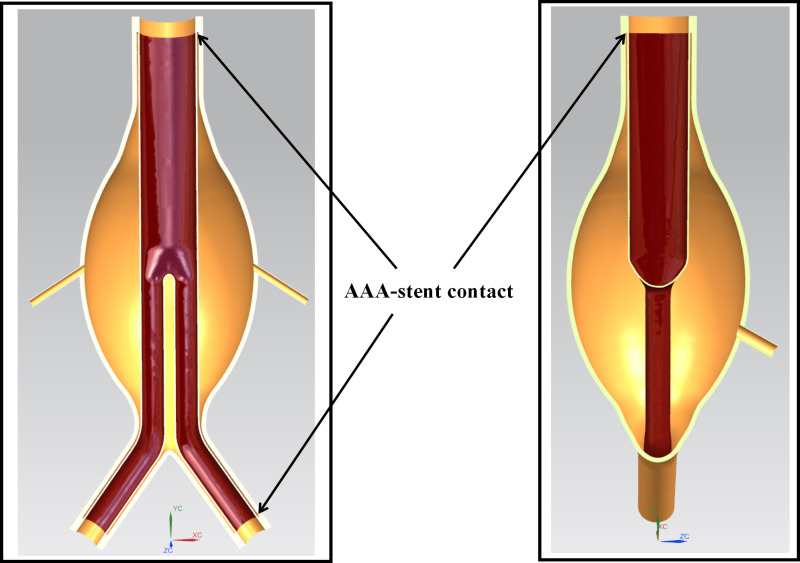
Type II endoleak simulation model.

This study developed a three-dimensional ideal model of a Type II endoleak, as shown in Fig 1. The model includes the AAA body, three branch arteries (the IMA and two LAs), and a stent graft. The stent graft is represented as a tubular structure with a thickness of 0.5 mm, without the stent mesh. Detailed geometric parameters are listed in Table 1.

**Table 1:** Geometric parameters of the AAA model with Type II endoleak.

Geometric Parameter	Value (mm)
Maximum Diameter of AAA	55
Inlet Diameter of AAA	20
Outlet Diameter of iliac artery	10
Diameter of IMA	4
Diameter of LA	2
Wall Thickness of AAA	1.5
Wall Thickness of IMA and LA	0.5
Stent Wall Thickness	0.5

Both the fluid and solid domains were meshed using tetrahedral elements. To enhance the accuracy of boundary layer flow calculations, five layers of boundary layer mesh were added in the fluid domain, and the mesh was refined in the branch artery regions. The total number of fluid domain mesh elements is 1,217,088, while the solid domain contains 606,139 elements. The mesh configuration is shown in S1 Fig.

### 2.2 Boundary conditions and numerical solution

**Fig 2:**
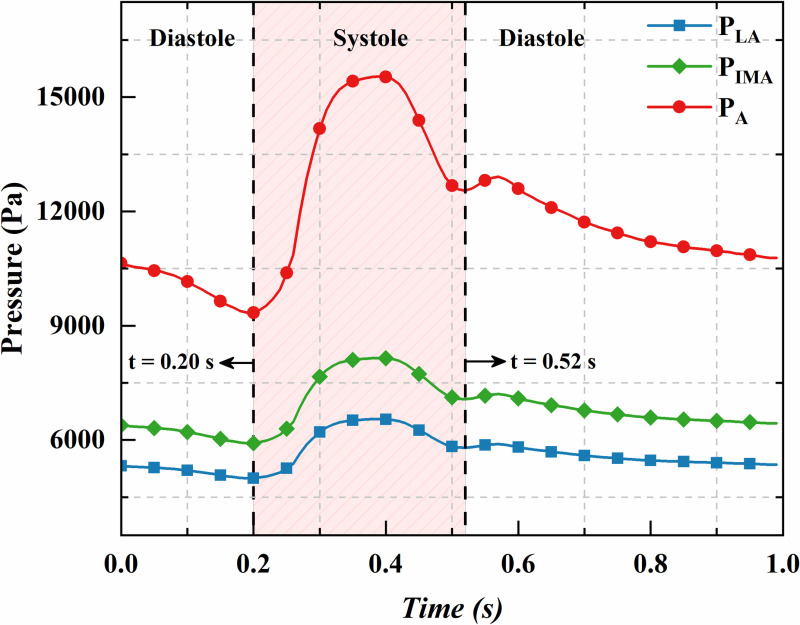
Standard arterial pressure and branch artery inlet pressure.

Currently, the methods for measuring the inlet boundary conditions of Type II endoleak primarily include ultrasound for measuring blood flow velocity and direct pressure measurements using pressure sensors during open surgery. However, ultrasound is often unable to accurately monitor blood flow velocity parameters in small branch vessels, and the limited number of open surgery cases makes it difficult to obtain relevant data. Thus, this study follows the assumptions from references [23,24], where the pressure waveform at the branch artery inlet is assumed to mirror the aortic waveform with a proportionally reduced amplitude. The aortic pressure (P_A_) waveform used in this study is shown in Fig 2 [25], where the systolic phase lasts from 0.20 to 0.52 seconds, and the mean aortic pressure is 89.2 mmHg. To clearly describe the inlet pressures of the branch arteries, the inlet pressure of the IMA is defined as P_IMA_, and that of the LA as P_LA_. During one cycle, the ratio of P_IMA_ to P_A_ is 0.6, while the ratio of P_LA_ to P_A_ is 0.5. Both P_IMA_ and P_LA_ have proportionally reduced amplitudes, as illustrated in Fig 2. The mean pressures of P_IMA_ and P_LA_ are 51.3 mmHg and 42.2 mmHg, respectively.

Notably, in this study, pressure is used to define the boundary conditions for each branch vessel, without imposing any restrictions on blood inflow or outflow. In other words, blood flow direction in each branch artery depends on the pressure difference between the inlet and the sac. Blood flows into the branch artery when inlet pressure exceeds sac pressure; otherwise, it flows out. This setup closely resembles real clinical conditions.

In this study, blood is treated as a homogeneous, incompressible non-Newtonian fluid with a density of 1060 kg/m³, modeled according to the Carreau-Yasuda viscosity model [26,27]. The vessel wall is considered an isotropic, linear elastic material, with properties including an elastic modulus of 2.7 MPa, Poisson’s ratio of 0.45, and a density of 1130 kg/m³. The stent is represented as isotropic structural steel, characterized by an elastic modulus of 2 × 10^5^ MPa, a Poisson’s ratio of 0.3, and a density of 7850 kg/m³ [28–30]. Additionally, this model does not account for residual wall stresses, thrombus formation, or the influence of surrounding tissues.

Considering that one of the main focuses of this study is to compare the differences between CFD and FSI simulation methods, we utilized an idealized model to better control variables and avoid the unfavorable effects posed by the complex geometry of patient-specific models, which can increase the difficulty of achieving convergence in two-way fluid structure interaction and reduce the reliability of computational results.

Simulations were conducted for three consecutive cardiac cycles in this study for both CFD and FSI cases, with data from the final cycle used for analysis. Each simulation used a time step of 0.01 seconds. The bidirectional FSI process was repeated until the differences in displacement and force between the last two iterations were below 1%. To balance computational accuracy and resource efficiency, the number of iterations per time step ranged from 5 to 100 to ensure convergence. Velocity-pressure coupling was handled using the Pressure-Implicit with Splitting of Operators (PISO) scheme. The finite element method (FEM) modeled interactions among blood, the vessel wall, and the stent. The fluid simulation settings for both CFD and FSI cases were identical.

### 2.3 Governing equations

#### 2.3.1 Fluid governing equations.

In this study, the continuity and momentum equations for blood flow are presented in (1) and (2):


∇·u=0 (1)

$$\rho \left(\frac{\partial u}{\partial t}+u\cdot \nabla \right)=-\nabla P+\mu \nabla^{2}u$$ (2)

Here, ***u*** represents velocity, *P* is static pressure, *ρ* is fluid density, and *μ* is dynamic viscosity.

Due to the deformation and aggregation of red blood cells, the shear strain rate and stress in blood flow exhibit nonlinearity, making blood essentially a non-Newtonian fluid. In this study, the Carreau-Yasuda model [27] is employed to describe the non-Newtonian properties of blood, which is widely used in blood flow analysis. The model is expressed as follows:


$$\eta (\dot{\gamma })=\eta_{\infty }+(\eta_{0}-\eta_{\infty }){\left(1+{\left(\lambda \dot{\gamma }\right)}^{a}\right)}^{\frac{n-1}{a}}$$ (3)

Here, $\text{η}\left(\dot{\text{γ}}\right)$ represents the fluid viscosity, $\dot{\text{γ}}$ is the local shear rate, with ${\text{η}}_{\text{∞}}$ and ${\text{η}}_{\text{0}}$ being the viscosities at infinite and zero shear rates, respectively (i.e., the limiting viscosity of the fluid at extremely high shear rates and the static viscosity at extremely low shear rates). *λ* is the relaxation time, describing the time required for the fluid to transition from infinite to zero shear rate viscosity. *n* is the flow behavior index, indicating the degree of shear thinning or thickening of the fluid, and *a* is a parameter that adjusts the shape of the shear rate curve’s transition region, known as the Yasuda index. The variation curve of blood viscosity with respect to strain rate is shown in S2 Fig.

#### 2.3.2 Solid governing equations.

The equilibrium equation relating strain and stress in the solid domain is expressed as follows:


$$\rho_{s}\frac{\partial^{2}u}{\partial t^{2}}-\nabla \cdot \sigma =\rho_{s}b$$ (4)

where *ρ*_*s*_ is the solid density, ***u*** represents the solid displacement, ***b*** is the volume force acting on the structure, and *σ* is the Cauchy stress tensor.

In this study, the vascular wall is assumed to be a linear elastic material. For an isotropic linear elastic solid, the stress tensor is expressed as follows:


$$\sigma =2\mu_{L}\epsilon +\lambda_{L}\text{tr}(\epsilon )I$$ (5)

where *λ*_*L*_ and *μ*_*L*_ are the first and second Lamé parameters, respectively, ϵ is the strain tensor, tr denotes the trace function, and *I* is the identity matrix.

For compressible materials, the Lamé parameters can be expressed in terms of Young’s modulus E and Poisson’s ratio *v* as follows:


$$\lambda_{L}=\frac{vE}{\left(1+v)(2v-1\right)}$$ (6)

$$\mu_{L}=\frac{E}{2(1+v)}$$ (7)

#### 2.3.3 Liquid-solid interface treatment.

In two-way fluid-structure interaction, the fluid’s action causes the solid domain to deform, and this deformation, in turn, alters the fluid-solid interface. As a result, the fluid flow equations must be expressed using fluid variables that move relative to the grid. This study applies the Arbitrary Lagrangian-Eulerian (ALE) method to viscous incompressible fluids, modifying the Navier-Stokes momentum equations as follows:


$$\rho \left(\frac{\partial u}{\partial t}+\left((u-u_{g})\cdot \nabla \right)u\right)=-\nabla p+\mu \nabla^{2}u$$ (8)

Here, ***u*** represents the fluid velocity, and ***u***_*g*_ is the velocity of the moving grid. In the ALE formulation, the term (***u*** *-* ***u***_*g*_) is added to the traditional Navier-Stokes equations to account for the grid movement.

The velocity and stress at the interface are shown in (9) and (10):


$$u_{f,\Gamma }=u_{s,\Gamma }$$ (9)

$$t_{f,\Gamma }=t_{s,\Gamma }$$ (10)

Here, ${\text{u}}_{\text{f,\textbackslash{}Gamma}}$ represents the fluid displacement at the interface, and ${\text{u}}_{\text{s,\textbackslash{}Gamma}}$ represents the solid displacement at the interface. ${\text{t}}_{\text{f,\textbackslash{}Gamma}}$ is the fluid force, and ${\text{t}}_{\text{s,\textbackslash{}Gamma}}$ is the solid force at the interface. The velocity continuity condition ensures consistent motion between the fluid and solid at the interface, meaning there is no relative velocity difference. The stress continuity condition ensures force equilibrium at the interface.

## 3 Results and discussion

### 3.1 Velocity and volume flow rate

**Fig 3:**
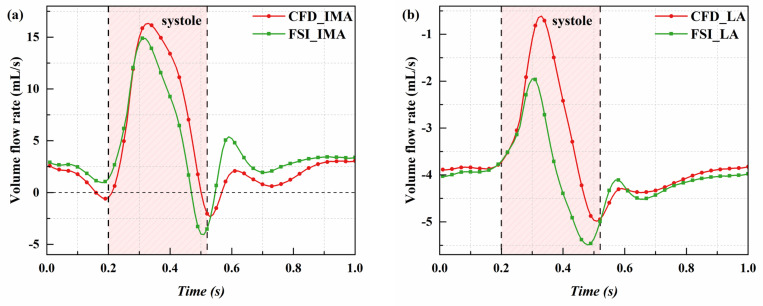
Comparison of CFD and FSI results: variation of flow rate in the IMA and LA over time. (a) Flow rate in the IMA; (b) Flow rate in the LA.

The volume flow rate and velocity serve as indicators of the leakage volume in Type II endoleaks, with higher values reflecting more severe endoleaks and greater challenges in thrombus formation within the sac. Fig 3 and S3 Fig show the temporal variations in volume flow rate and flow velocity in the IMA and LA under pressure, analyzed using both CFD and FSI methods. The results reveal that, during most of the cardiac cycle, blood enters the sac through the IMA and exits via the LA, forming the typical pattern of endoleak. However, it is noteworthy that there are moments when the volume flow rate and pressure difference in the IMA become negative, suggesting that blood flow is not strictly unidirectional through specific branch vessels. Instead, it depends on pressure differences between the sac and branch vessels.

The CFD and FSI results show complete agreement in trends but differ quantitatively. CFD predictions lag slightly behind FSI temporally, with higher flow rates and velocities during systole but similar or lower values during diastole. In particular, the peak flow rates predicted for the IMA by CFD and FSI are 16.33 mL/s (at 0.33 s) and 14.90 mL/s (at 0.31 s), respectively, with a difference of 10.17%. The peak flow rate difference for the LA is 9.20%. Additionally, when the LA outflow velocity is at its minimum, CFD predicts -0.61 mL/s, while FSI predicts -1.95 mL/s, a difference of 68.72%.

These two methods, CFD and FSI, differ fundamentally in their approaches to modeling blood flow. CFD directly converts pressure differentials into blood flow velocities, whereas FSI accounts for the deformation of the vessel wall. This deformation not only reduces the blood flow velocity but also causes velocity peaks to occur earlier. Another critical factor influenced by FSI is the change in the effective flow area due to vessel wall deformation. Specifically, during systole, vessel expansion increases the effective flow area, which in turn reduces the blood flow velocity. Conversely, during diastole, vessel contraction decreases the effective flow area, thus increasing the blood flow velocity. These dynamics underscore the importance of incorporating fluid-structure interactions in predictions of flow velocities, particularly in scenarios demanding high accuracy.

The overestimation of peak velocity by the CFD method may affect the evaluation of the leakage volume in endoleak branch vessels and the assessment of potential thrombus formation risks. CFD results tend to suggest that thrombus formation after EVAR is more challenging, thereby increasing the risk of Type II endoleak. This could potentially lead to overtreatment, such as embolization of unnecessary branch vessels, resulting in complications like intestinal ischemia or spinal cord ischemia. This highlights the importance of selecting an appropriate simulation method when assessing the risk of Type II endoleak.

S4 Fig shows the streamline and velocity contour plots on the central plane at the peak velocity moment under the FSI method, illustrating the flow pattern associated with endoleak formation. At its peak, the velocity in the IMA reaches approximately 1.18 m/s, creating a high-speed jet of blood that enters the aneurysmal sac and forms a confined jet within the sac. The velocity gradient between the jet and surrounding blood creates shear forces, producing vortices on both sides and generating localized blood flow circulation. Upon impacting the stent, the jet deflects and quickly dissipates. During outflow through the LA, blood within the aneurysmal sac gradually converges toward the outlet, accelerating to a peak outflow velocity of around 0.87 m/s. Comparison of CFD and FSI simulation results shows no significant difference in flow patterns between the two approaches.

Overall, high-velocity regions within the sac are mainly concentrated around the inlet and outlet vessels and in their downstream impact zones, while most of the aneurysmal sac remains a low-velocity area (velocity <0.01 m/s). This suggests that blood inflow and outflow through the IMA and LA fail to sufficiently mix the blood within the sac, which may promote clotting and thrombus formation. Additionally, due to blood’s non-Newtonian properties, regions of lower flow velocity exhibit higher viscosity, making them more resistant to movement, further contributing to thrombus formation.

### 3.2 Aneurysmal Sac Pressure

Sac pressure is a critical parameter for evaluating the prognostic success of EVAR procedures. To investigate whether sac pressure varies across different spatial locations within the aneurysm sac, we strategically placed three sampling points. The pressure variation curves demonstrated high consistency, as pressure within the sac transmits rapidly and uniformly (see S5 Fig). Consequently, a single sampling point was selected to represent the whole sac pressure.

**Fig 4:**
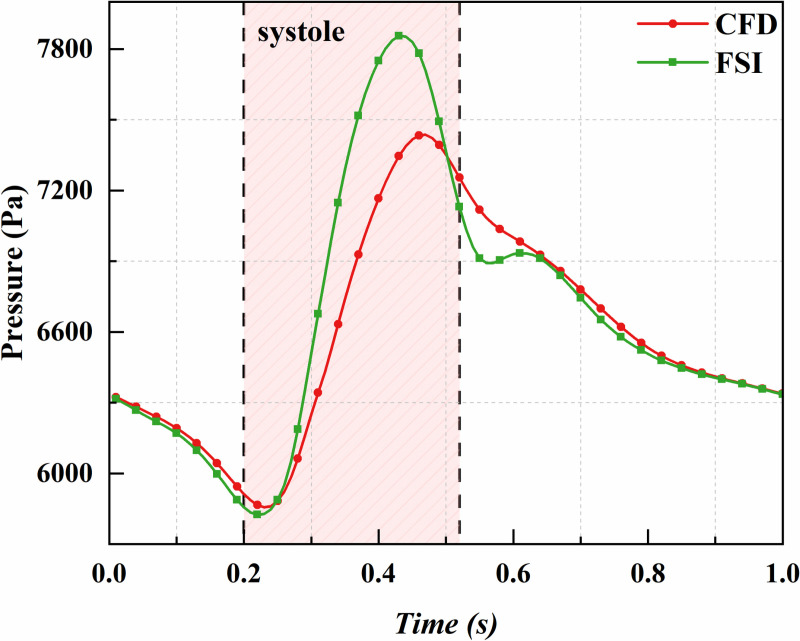
Comparison of CFD and FSI results: variation of sac pressure over time.

Fig 4 presents the CFD and FSI predictions for mean sac pressure over a cardiac cycle. The FSI-predicted mean sac pressure is significantly higher than the CFD prediction, with a peak increase of 5.33%. This difference is attributed to the expansion of the vessels and sac in the FSI simulation during the cardiac cycle, which increases the effective flow area and lowers flow velocity. Since blood flow is incompressible and follows Bernoulli’s principle (under negligible gravity and flow losses), a decrease in velocity corresponds to an increase in pressure. Additionally, the FSI-predicted mean sac pressure aligns more closely with the branch vessel inlet pressures (Figs 2 vs. 4), whereas the CFD model omits some of these nuanced details.

In previous studies, Zhang et al. [31] found that residual sac pressure exceeding 50 mmHg could lead to the continued expansion of an aneurysm, while pressure below this threshold might promote aneurysm shrinkage. In this study, the FSI method predicted a mean sac pressure of 6654.2 Pa (49.9 mmHg) and a peak pressure of 7855.4 Pa (58.9 mmHg), whereas the CFD method predicted a mean sac pressure of 6590.5 Pa (49.4 mmHg) and a peak pressure of 7436.5 Pa (55.8 mmHg). Notably, the mean sac pressure predicted by the FSI method is very close to the 50 mmHg threshold suggested by Zhang et al., while the CFD method slightly underestimates it by approximately 1%.

Both CFD and FSI methods yield similar results for the average sac pressure difference. The main discrepancy between the two methods lies in the maximum value and the fluctuation over a cardiac cycle. The CFD approach may underestimate the fluctuations in sac pressure. For patients with sac pressures near the critical threshold of 50 mmHg, the use of the FSI method is recommended to better predict both the maximum pressure and the fluctuations in sac pressure.

**Fig 5:**
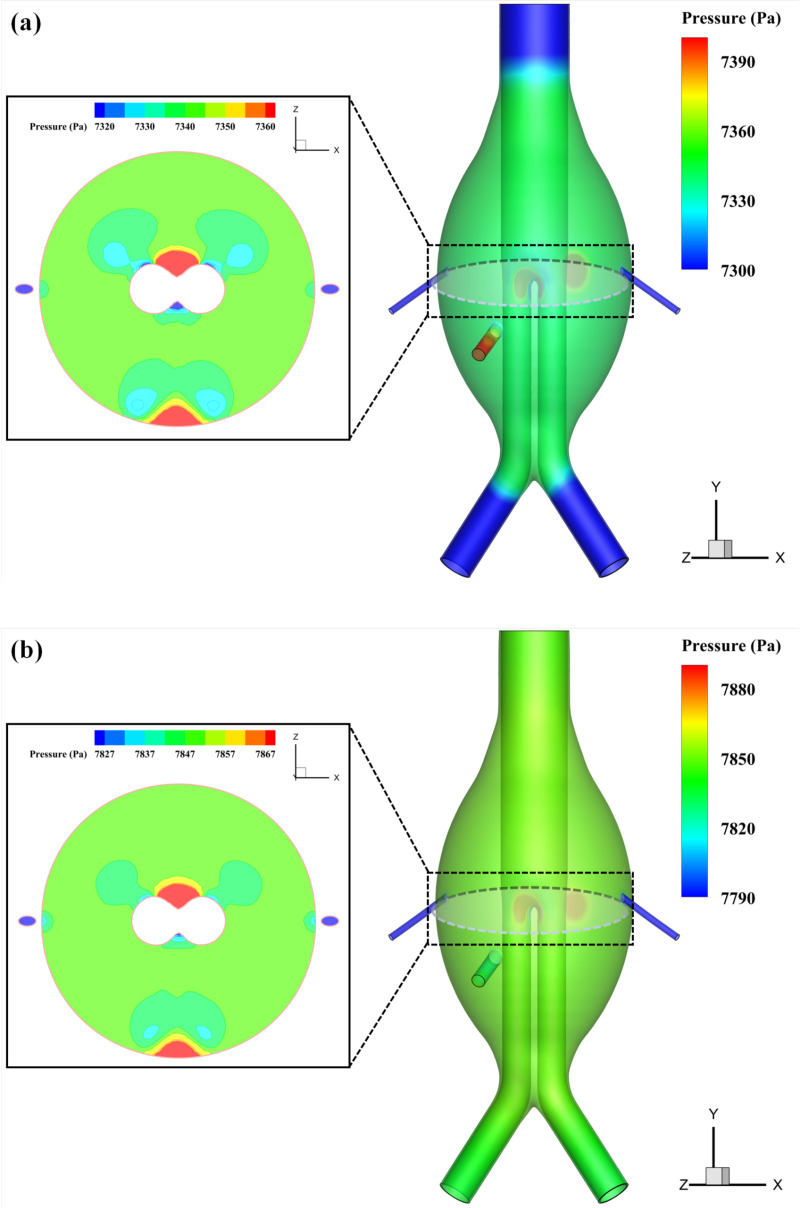
Comparison of CFD and FSI results: pressure distribution inside the aneurysmal sac at the time of peak pressure. (a) CFD method; (b) FSI method.

The distribution of static pressure reflects the forces exerted on the sac wall and stent. Fig 5 presents the static pressure distribution within the aneurysmal sac at 0.43 s, when the sac pressure reaches its peak under the FSI method. The pressure distribution inside the sac appears relatively uniform; however, higher local pressures are observed at the bifurcation where blood flow from the IMA strikes the stent and the opposite sac wall. This indicates that these two areas experience greater forces, which may lead to potential stent displacement and significant deformation of the opposite sac wall.

Compared to the CFD method, the FSI results show a more uniform spatial pressure distribution, likely due to the FSI method’s consideration of fluid-structure interactions, which more accurately capture the impact of stent and sac wall deformations on pressure distribution. Additionally, at this moment, both methods predict LA inlet pressures lower than the actual sac pressure, a pressure difference that directly influences the direction of blood flow within the branch arteries, causing blood to flow out of the sac through the LA.

**Fig 6:**
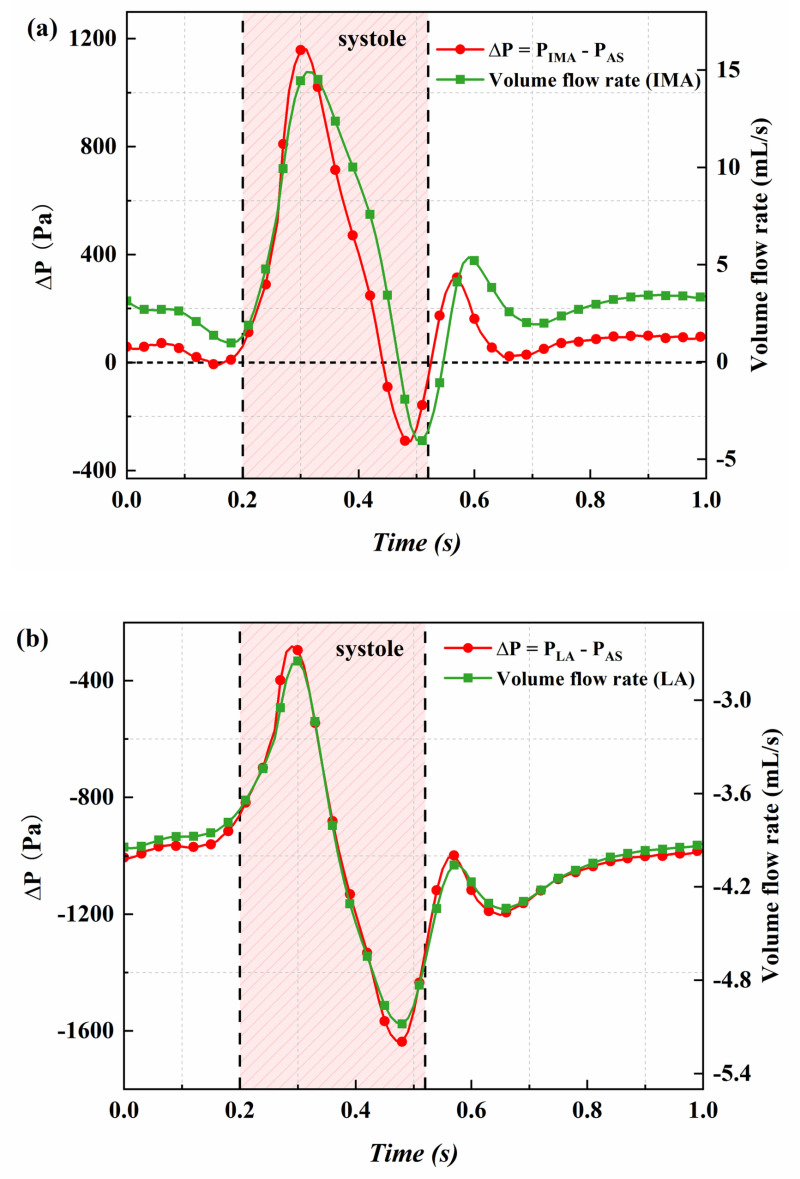
Comparison of ΔP and volume flow rate using the FSI method. (a) ΔP and IMA volume flow rate; (c) ΔP and LA volume flow rate.

To elucidate the mechanism of Type II endoleak, we defined ΔP as the pressure difference between the IMA inlet pressure and the sac pressure, or alternatively, between the LA inlet pressure and the sac pressure. Fig 6 illustrates the relationship between the volume flow rate through the branch vessels and ΔP, as determined by the FSI method. Corresponding results obtained from the CFD analysis are presented in S6 Fig. A strong correlation exists between ΔP and volume flow rate. Variations in volume flow rate do not follow the sac pressure waveform (Figs 2 and 3) but instead align more closely with changes in ΔP, indicating that the pressure difference between the branch vessels and the sac is the primary factor determining volume flow rate. A comparison of FSI and CFD results shows that CFD can capture the relationship between ΔP and volume flow rate, though the FSI predictions are more accurate. In clinical practice, if it is necessary to control the leakage volume, regulating the inlet pressure of the endoleak can be considered. This also highlights the importance of strict blood pressure management following EVAR.

### 3.3 Wall shear stress

**Fig 7:**
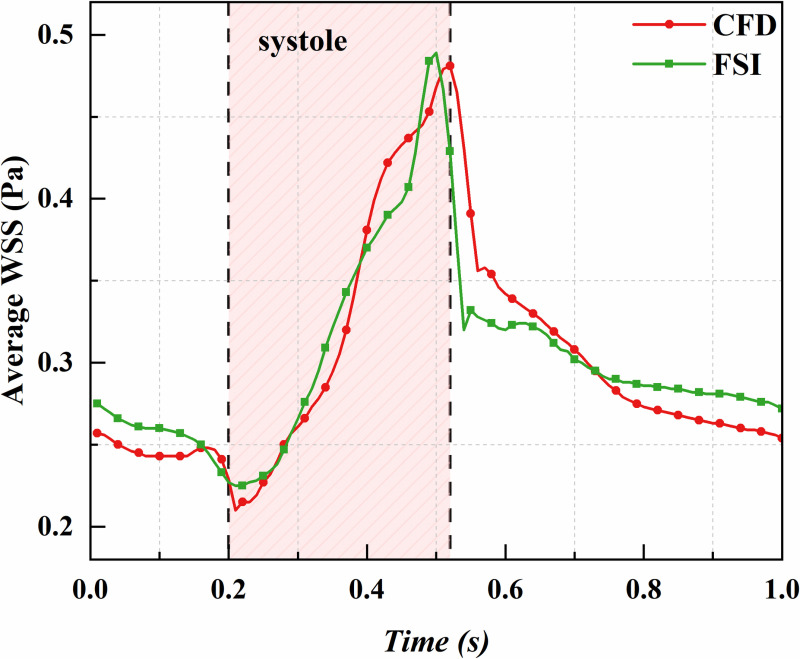
Comparison of CFD and FSI results: variation of average WSS over time.

WSS significantly influences the function and structure of vascular endothelial cells, making it critical for vascular health. It is crucial for regulating blood flow and is closely linked to the formation of atherosclerosis and the development of thrombosis. Both excessively high and low WSS can lead to vascular disease and compromise the structural integrity of the vessel wall. Fig 7 shows the predicted average WSS on the vessel wall from CFD and FSI analyses. While both methods show some temporal discrepancies, with CFD results slightly lagging, the quantitative differences within the cardiac cycle are minimal. Outside the cardiac cycle, CFD predictions tend to be slightly lower, with an overall difference of around 0.65%; however, the absolute slope of the CFD predictions is steeper than that of the FSI method. Therefore, if only the average WSS is of concern, the difference between the two methods is minimal, and the CFD method can be used to save computational time.

**Fig 8:**
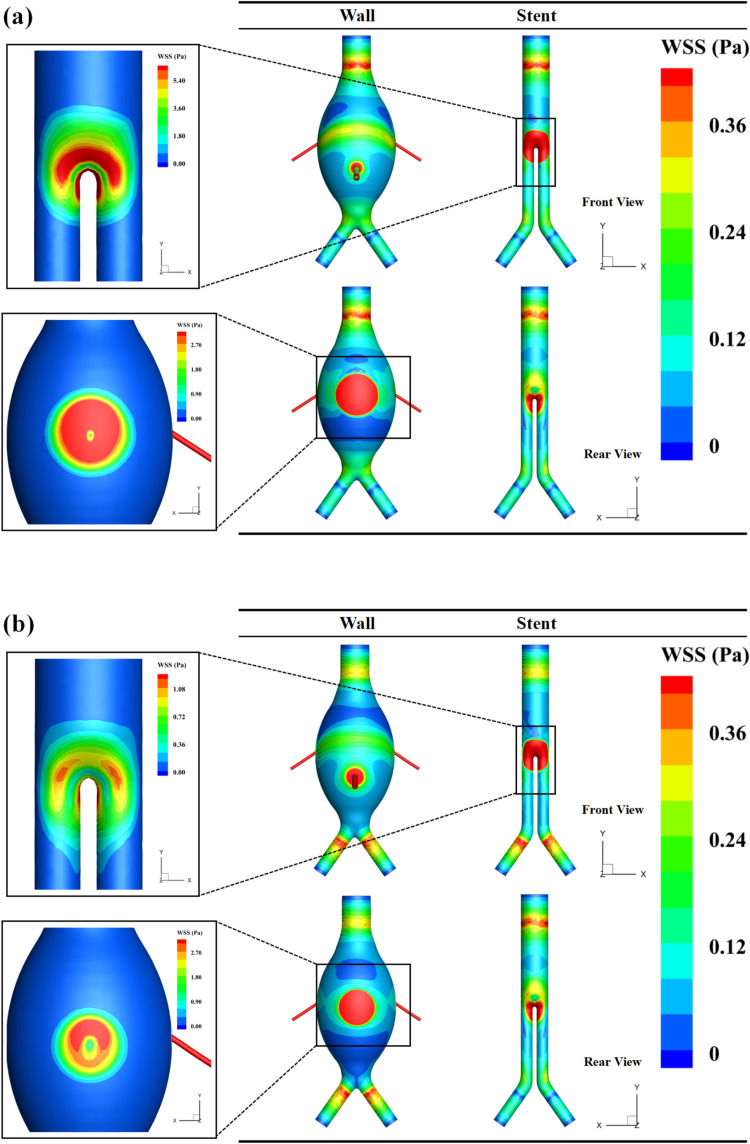
Comparison of CFD and FSI results: distribution of average WSS at the time of peak value. (a) CFD method; (b) FSI method.

Under the FSI method, the peak average WSS occurs at 0.49 s. Fig 8 shows the WSS contour maps from both CFD and FSI at this moment. As seen in the figure, WSS is generally low across the aneurysmal sac, with higher WSS values concentrated on the walls of the branch arteries, particularly near the bifurcation. This is caused by the impact of the IMA on this region. The FSI method provides a more detailed representation of the fluid-structure interaction in these areas.

## 4 Conclusions

In this study, an idealized model of Type II endoleak with three patent branch arteries was constructed, and CFD and FSI methods were used to analyze branch vessel flow velocity, leakage volume, sac pressure, and WSS. The key conclusions are as follows:

**1.** **Mechanism of Type II Endoleak:** Type II endoleak is caused by the pressure difference between the sac and the branch vessels. Clinically, the pressure in the IMA is often higher, making it the primary “culprit vessel” for endoleak.
**2.** **Flow Patterns in Type II Endoleak:** During endoleak, significant flow disturbances are confined to the areas near the IMA and LA, while most of the aneurysmal sac remains in a very low-velocity state, promoting thrombosis and coagulation. The non-Newtonian nature of blood — lower viscosity in flowing regions and higher viscosity in stagnant areas — further exacerbates clot formation in low-velocity zones.
**3.** **Comparison of CFD and FSI:** CFD can qualitatively predict flow patterns within the sac but tends to overestimate peak flow velocity by about 9.70% and underestimate sac pressure by approximately 5.33% due to the lack of modeling vessel wall deformation and the expansion-contraction effects of flow channels. Additionally, CFD results exhibit a temporal lag and some distortion in the velocity and pressure fluctuations across the cardiac cycle. In contrast, FSI provides more accurate predictions, better capturing the relationship between sac pressure and branch vessel inlet pressure. Clinically, CFD is recommended for rapid assessments, while FSI is more suitable for detailed mechanistic studies.

The findings of this study provide a biomechanical explanation for the mechanisms of Type II endoleak and the blood flow patterns associated with its occurrence, aiding clinical understanding. By quantitatively comparing the differences between CFD and FSI methods, this research also offers valuable insights to guide method selection in related studies within the field.

## 5 Limitations

Under the idealized assumptions, this study quantitatively compares the results of different numerical simulation methods (CFD and FSI), providing a preliminary theoretical framework for investigating the dynamic characteristics of Type II endoleak. However, it must be acknowledged that these idealized assumptions may affect the applicability of the conclusions in clinical practice. Therefore, future research should focus on further model optimization by incorporating patient-specific models to enhance the predictive capability and clinical relevance. In addition, we did not conduct sensitivity experiments, nor did we discuss their impact on the predictive accuracy in clinical contexts. Moreover, because accurate data on the blood flow velocity in branch arteries is difficult to obtain, this study did not compare the simulation results with real-world data. In future research, we will adopt clinical data to validate the reliability of the simulation methods.

## Supporting information

S1 Fig Mesh of Type II endoleak model. (TIF)

S2 Fig The relationship between fluid viscosity and strain rate for the Carreau-Yasuda Non-Newtonian fluid model. (TIF)

S3 Fig Comparison of CFD and FSI results: variation of velocity in the IMA and LA over time. (a) Velocity in the IMA; (b) Velocity in the LA.
(TIF)

S4 Fig Comparison of CFD and FSI results: streamlines and velocity contours on the central plane at the time of peak value. (a) ZY plane; (b) XY plane.
(TIF)

S5 Fig The pressure in the aneurysmal sac at different locations. (a) The pressure monitoring point locations; (b) the pressure variations against time for various monitoring point.
(TIF)

S6 Fig Comparison of ΔP and volume flow rate using the CFD method. (a) ΔP and IMA volume flow rate; (b) ΔP and LA volume flow rate.
(TIF)
