# Supplementary figures and images for: Numerical study on the biomechanics mechanism of Type II endoleak after EVAR for Abdominal Aortic Aneurysm
Source: PLoS One. 2025 May 28;20(5):e0323358. doi: 10.1371/journal.pone.0323358 (PMC12118978; doi:10.1371/journal.pone.0323358)

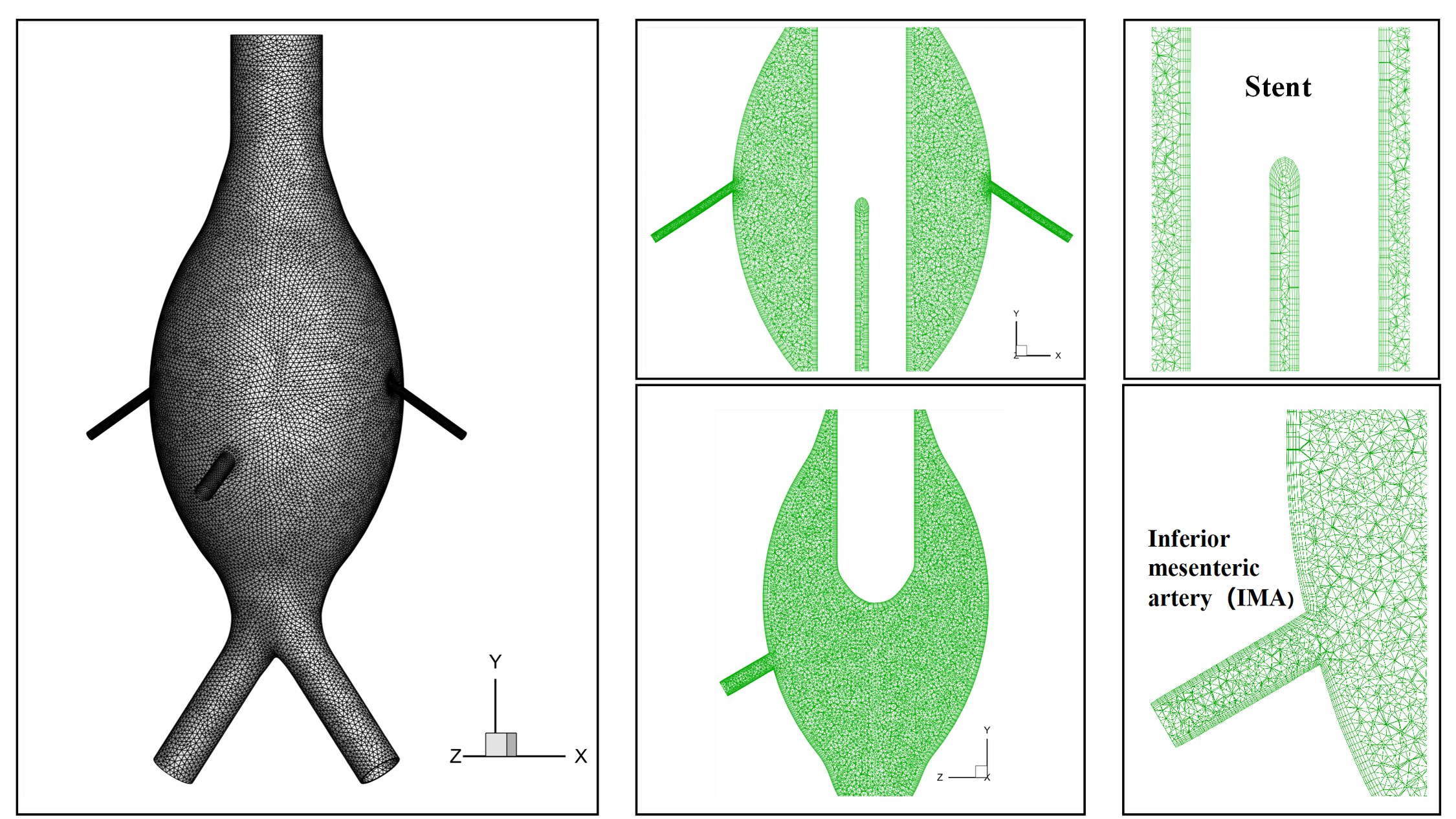

Supplement: S1 Fig — (TIF) [file pone.0323358.s001.tif]

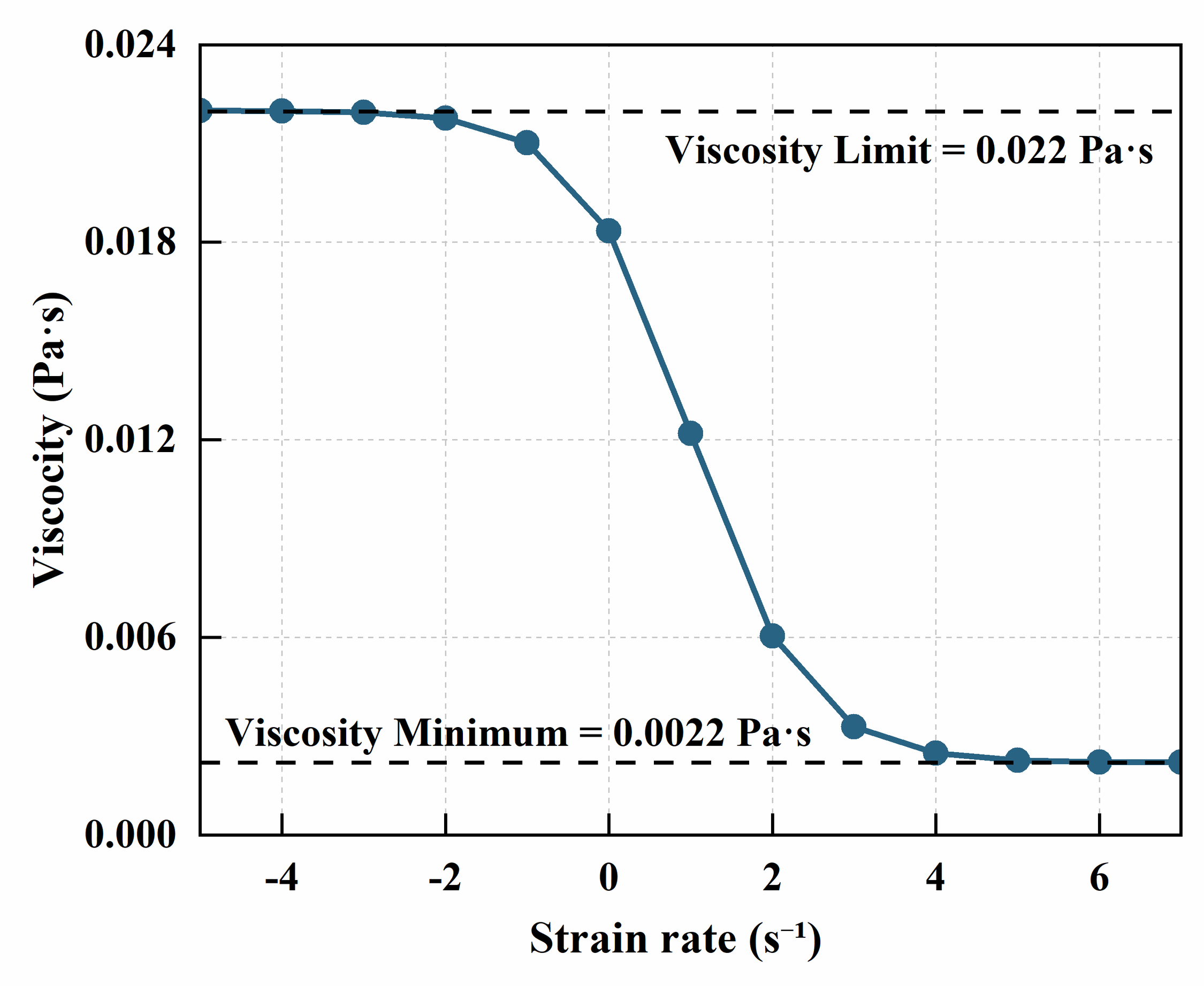

Supplement: S2 Fig — (TIF) [file pone.0323358.s002.tif]

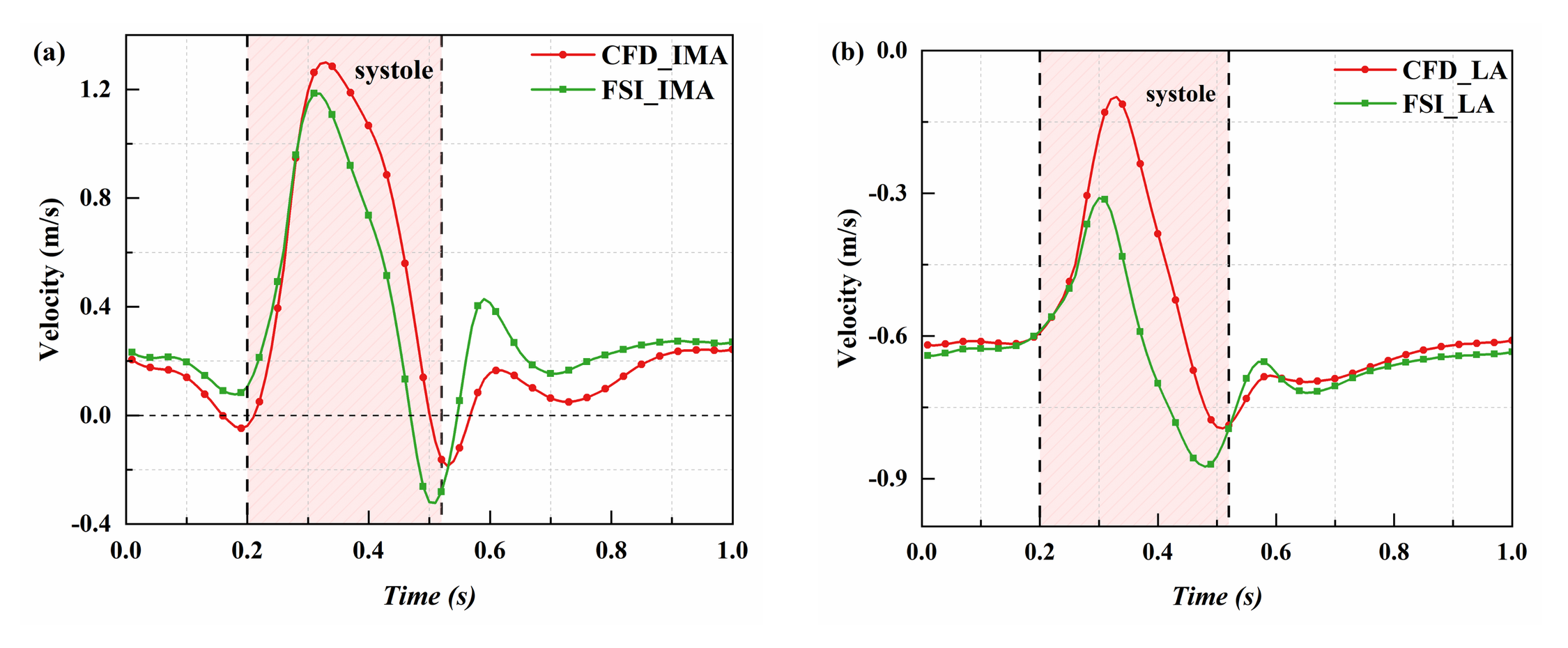

Supplement: S3 Fig — (a) Velocity in the IMA; (b) Velocity in the LA. (TIF) [file pone.0323358.s003.tif]

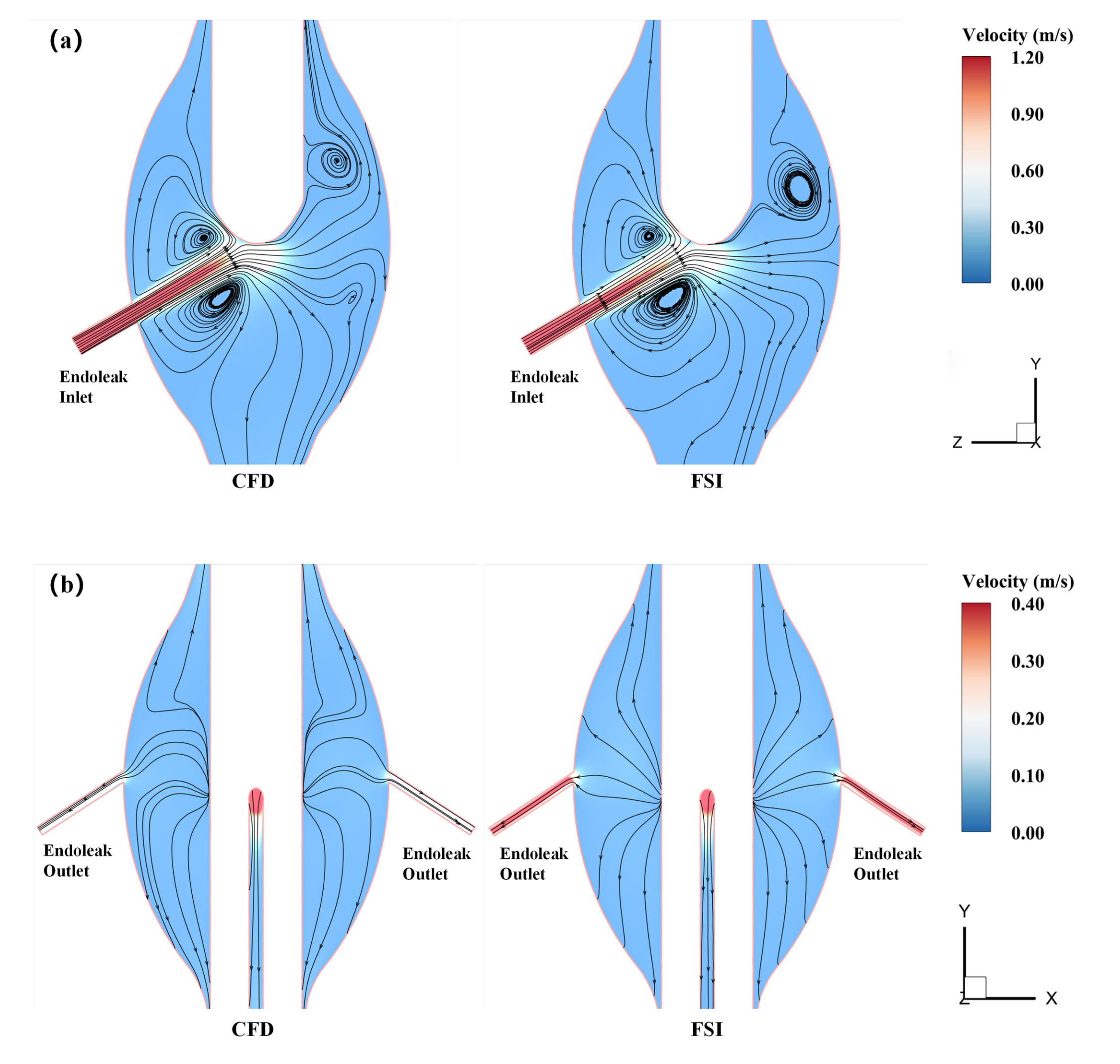

Supplement: S4 Fig — (a) ZY plane; (b) XY plane. (TIF) [file pone.0323358.s004.tif]

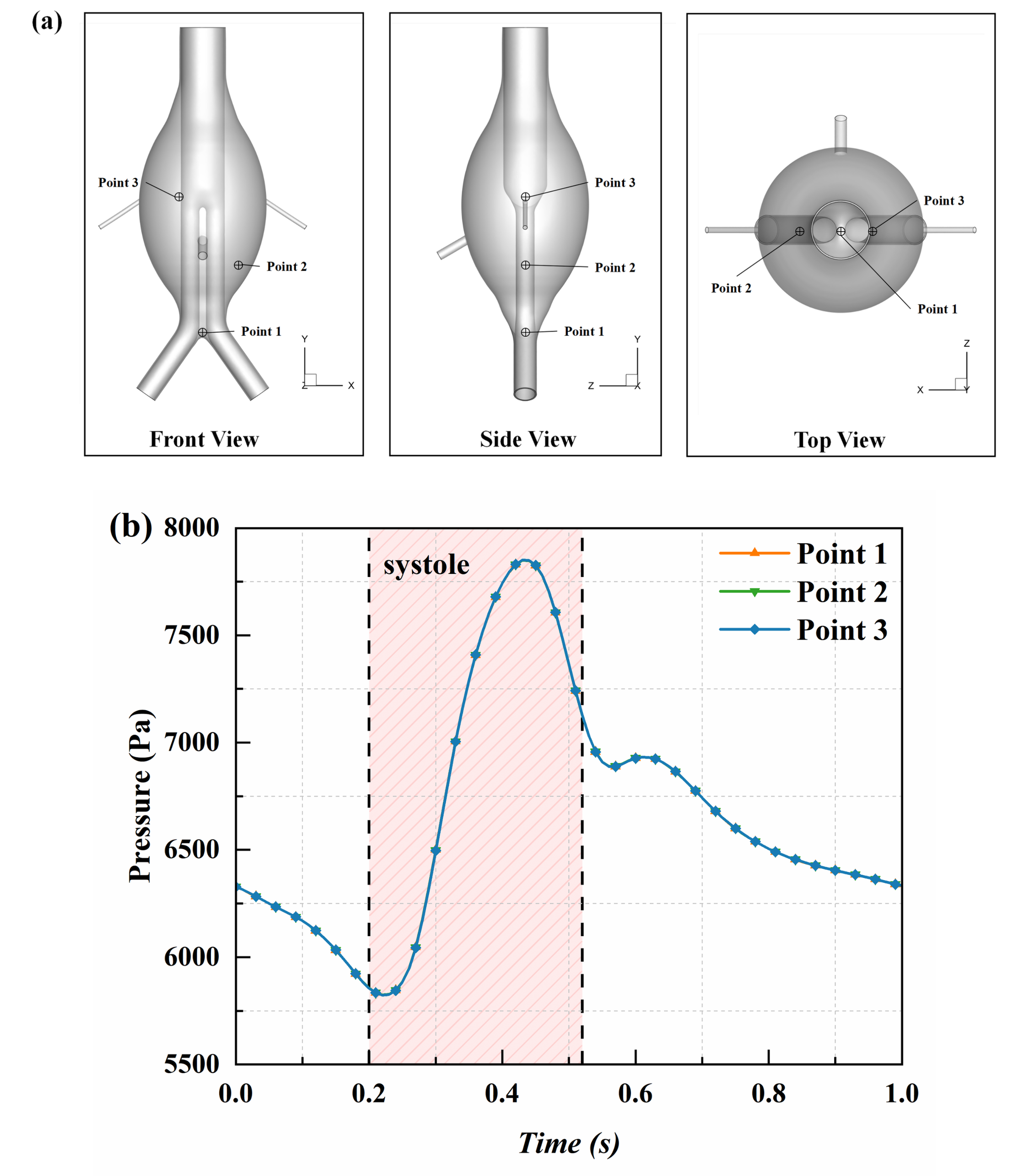

Supplement: S5 Fig — (a) The pressure monitoring point locations; (b) the pressure variations against time for various monitoring point. (TIF) [file pone.0323358.s005.tif]

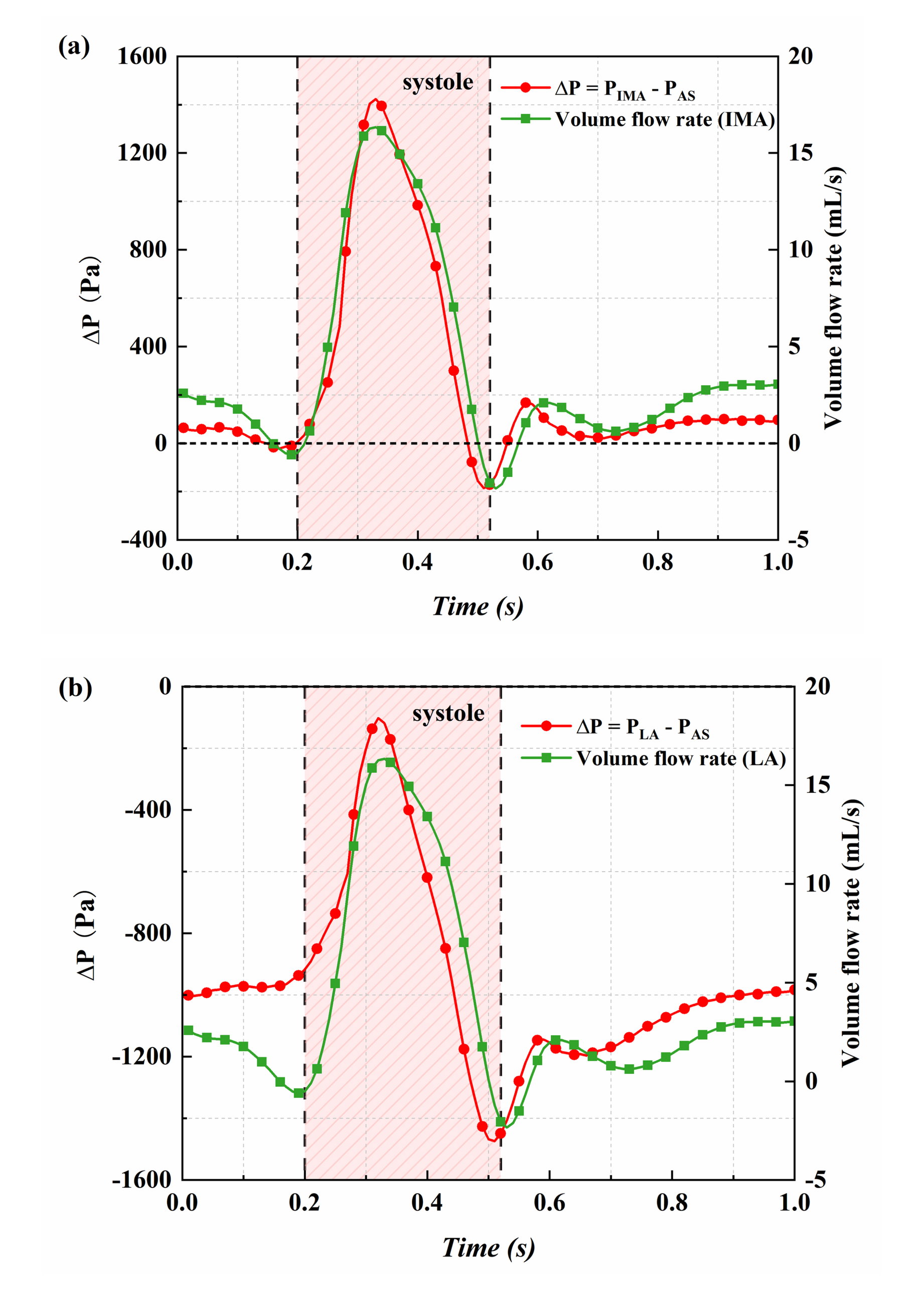

Supplement: S6 Fig — (a) ΔP and IMA volume flow rate; (b) ΔP and LA volume flow rate. (TIF) [file pone.0323358.s006.tif]
